# Atypical Neuroimaging Findings in a Patient With Mucopolysaccharidosis Type VII (Sly Syndrome)

**DOI:** 10.7759/cureus.66819

**Published:** 2024-08-13

**Authors:** Purnachandra K Lamghare, Urvashi Agarwal, Tushar Kalekar, Karishma S Krishnani

**Affiliations:** 1 Radiodiagnosis, Dr. D. Y. Patil Medical College, Hospital & Research Centre, Dr. D. Y. Patil Vidyapeeth (Deemed to be University), Pune, IND

**Keywords:** autosomal recessive, mri, sly syndrome, neuroimaging, mucopolysaccharidosis type vii

## Abstract

Mucopolysaccharidosis (MPS) consists of a heterogeneous group of multisystem disorders that are usually inherited. This spectrum consists of seven subtypes in total. Sly syndrome, also known as type VII MPS, is a multisystem disorder with a wide array of symptoms that overlap with other mucopolysaccharide disorders. Diagnosis of Sly syndrome relies on metabolic and radiological criteria. This report presents a case of a 19-year-old male who presented with seizures as his chief complaint. By metabolic workup done previously, he was diagnosed with Sly syndrome, an autosomal recessive mucopolysaccharide syndrome. This case underscores various multisystem features associated with the disease; however, it mainly highlights and emphasizes the diverse neurological features, including typical and atypical neuroimaging in Sly syndrome, aiding in its characterization, early diagnosis, and management.

## Introduction

Sly syndrome refers to a group of rare autosomal recessive genetic disorders characterized by lysosomal storage dysfunction. This disorder results in impaired degradation of mucopolysaccharides or glycosaminoglycans due to a deficiency in β-glucuronidase activity [1]. This enzyme is responsible for cleaving terminal glucuronic acid residues from dermatan sulfate, heparan sulfate, and chondroitin sulfate. The deficient activity of β-glucuronidase leads to the accumulation of these substances within the lysosomes of affected cells [2]. Mucopolysaccharidosis (MPS) VII, a subtype of Sly syndrome, occurs with an incidence of approximately one in 250,000 [3]. Mucopolysaccharidosis disorders typically involve multiple organ systems, and radiology plays a crucial role in diagnosing, evaluating complications, and assessing the progressive involvement of the brain, visceral organs, and skeletal system [4]. In Sly syndrome, medical imaging is particularly important for identifying neuroimaging features such as brain atrophy, thickened meninges, hydrocephalus, and spinal cord changes [5], which can lead to further complications. The abnormal accumulation of glycosaminoglycans can manifest clinically with hepatomegaly, splenomegaly, skeletal dysostosis, central nervous system changes, and otorhinolaryngological disorders [6-9]. This case report emphasizes the role of neurological imaging in the diagnosis and management of Sly syndrome.

## Case presentation

A 19-year-old male was brought to the hospital after a recent episode of seizures. Initial patient history revealed that the patient had been experiencing episodes of generalized tonic-clonic seizures (GTCS) since the age of nine. These episodes presented as tonic posturing of limbs, profuse sweating, and urinary incontinence. There was also associated cyanosis of the lips and tongue, followed by loss of consciousness and post-ictal confusion. However, there was no history of anti-epileptic drug use. The patient had a history of previous complaints including breathlessness, cough, and apneic spells. There was no family history of epilepsy or metabolic syndrome.

On physical examination, the patient exhibited coarse facial features, including macroglossia, malocclusion (suggestive of dentofacial deformity with mandibular prognathism), anterior open bite, a prominent forehead, hearing loss, and corneal clouding. The patient was noted to be of short stature relative to his age.

The patient also complained of snoring; however, the respiratory examination was normal. Neurological examination, including motor, sensory, and cranial nerve assessments, was normal. There was no history of developmental delay or psychological disabilities. The patient had no significant birth history or prior use of anti-epileptic drugs.

**Figure 1:**
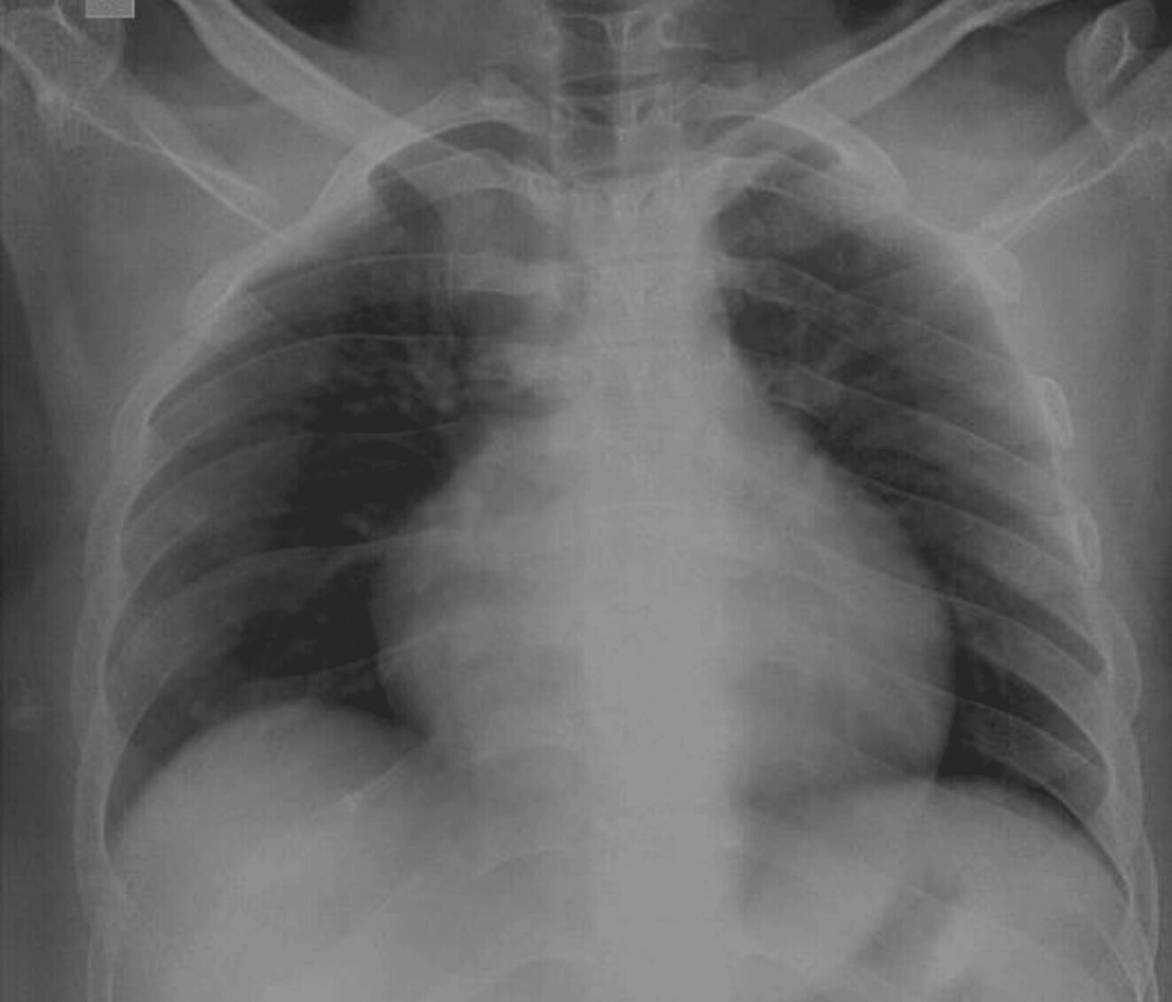
AP view of the chest X-ray shows cardiomegaly AP: Anteroposterior.

Routine hematological investigations, liver function tests (LFT), renal function tests (RFT), and thyroid-stimulating hormone (TSH) were all within normal ranges. However, an ultrasound scan revealed hepatomegaly and splenomegaly. A chest X-ray showed mild cardiomegaly (Figure 1), prompting a 2D echocardiography. The echocardiogram revealed myxomatous affection of the aortic and mitral valves with grade II mitral regurgitation and mild aortic regurgitation.

Subsequently, the patient underwent cardiac magnetic resonance imaging (MRI), which revealed a non-dilated left ventricle (LV) with preserved left ventricular ejection fraction (LVEF), early dilation of the right ventricle (RV) with reduced right ventricular ejection fraction (RVEF), and RV hypertrophy with increased trabeculation.

**Figure 2:**
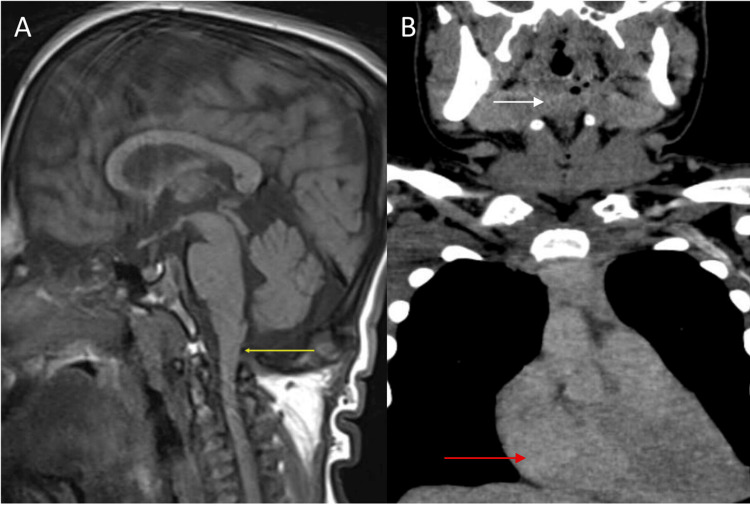
(A) Sagittal T1-weighted image (T1WI) shows crowding of the foramen magnum (yellow arrow) with the abnormal shape of cervical vertebrae, leading to spinal cord compression and compromise. (B) Coronal view shows soft tissue density (white arrow) causing oropharynx and hypopharynx narrowing. The red arrow indicates cardiomegaly.

Contrast-enhanced computed tomography (CECT) of the neck revealed bilateral adenoid hypertrophy, which was causing the narrowing of the retropharyngeal airways. Additionally, soft tissue density thickening was noted in the pre-vertebral region extending from C5 to D3 vertebrae levels, compromising the airways in the hypopharynx, nasopharynx, and oropharynx. The coronal section also revealed mild cardiomegaly (Figure 2).

**Figure 3:**
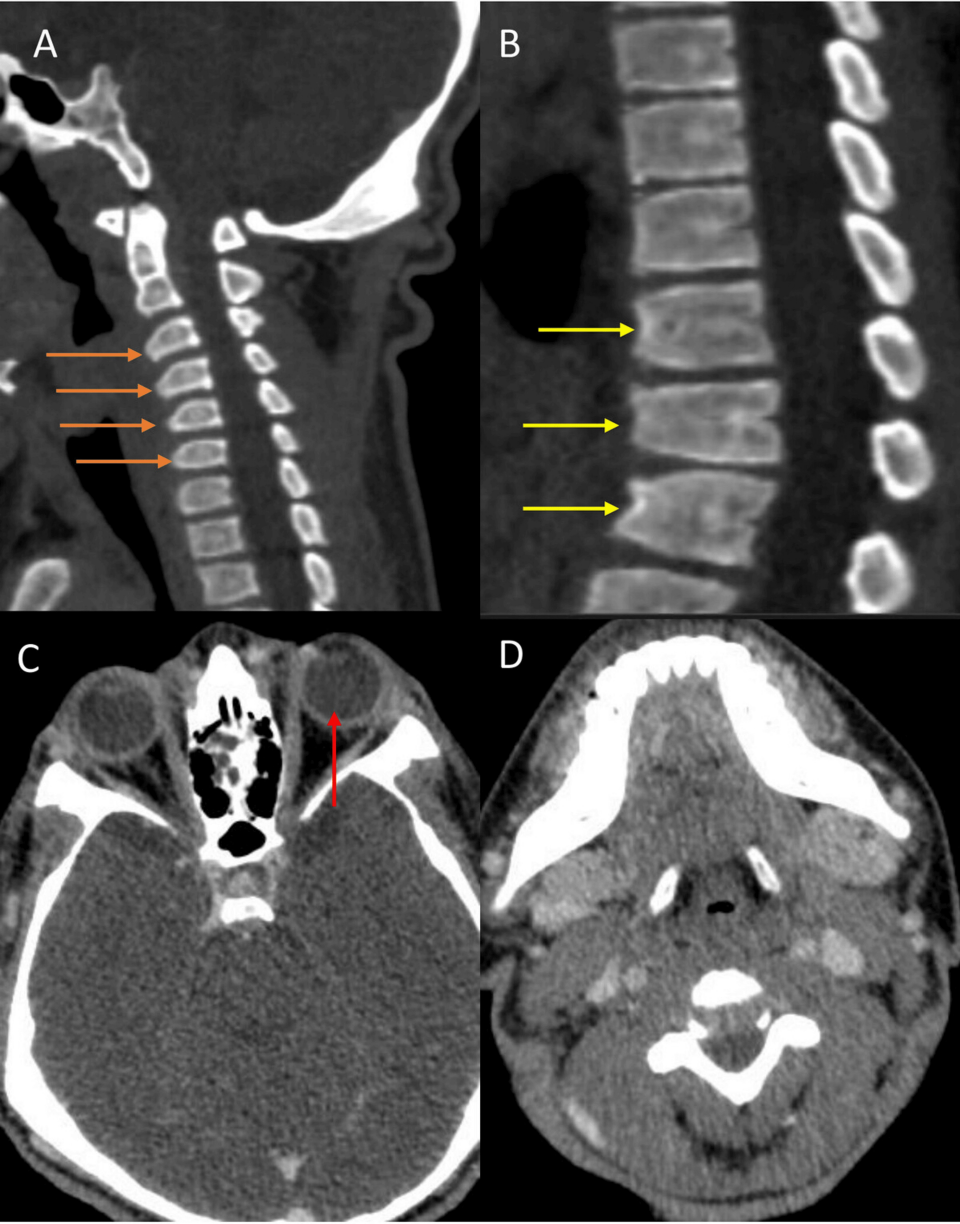
The CT sagittal view shows (A) the abnormal bullet shape of cervical vertebrae (orange arrows) causing spinal compromise and (B) the abnormal shape of thoracic vertebrae (yellow arrows). The axial CT section of the brain reveals (C) hypertelorism (red arrow) and (D) abnormal craniofacial bones with a short body of the mandible.

The bone window also revealed spinal canal compromise at the CV junction region with mild cord compression, which was attributed to abnormal bullet-shaped cervical vertebrae (Figure 3). Abnormal shapes of craniofacial bones, including a short body of the mandible, abnormal angulation, and hypoplasia of mandibular condyles contributed to an abnormal face shape. Thickening of both frontal and occipital bones, external protuberance, and mild hypotelorism were also noted (Figure 3).

**Figure 4:**
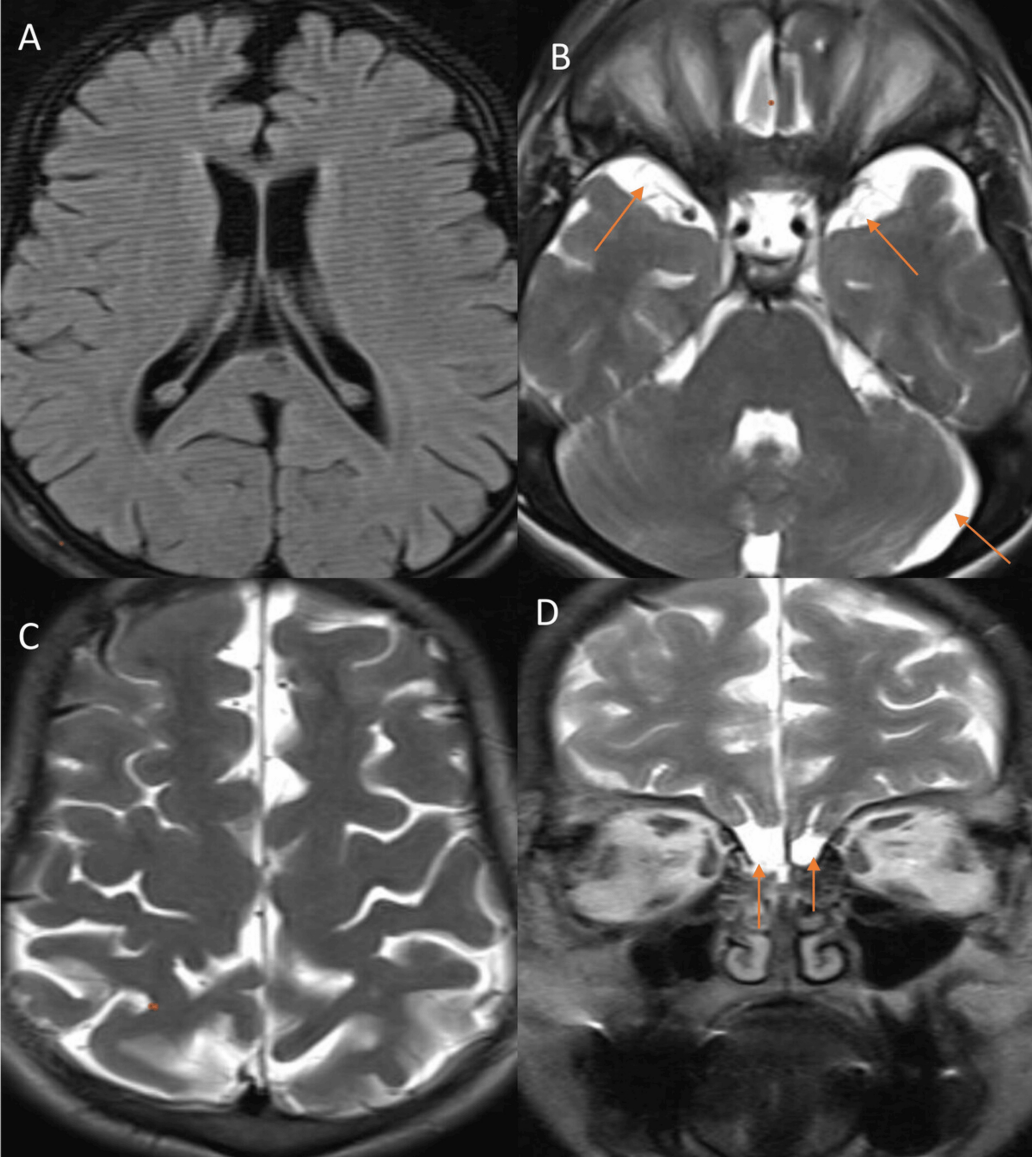
(A-C) Axial sections of the brain. (A) T1-weighted image (T1WI) shows prominent cortical sulci and ventricular system, suggestive of generalized cerebral brain atrophy. (B) T2-weighted image (T2WI) shows prominent subdural cerebrospinal fluid (CSF) spaces in the temporal region and left cerebral hemisphere (red arrows). (C) Craniosynostosis shows increased anteroposterior diameter and reduced transverse diameter, resulting in dolichocephaly. (D) The coronal section shows atrophy of the olfactory bulbs (red arrows).

Further, the patient underwent an MRI of the brain using a 1.5 Tesla Siemens machine (Siemens, Munich, Germany). Axial, coronal, and sagittal sections were acquired using the following sequences: T1-weighted (Fa 25, Te 4.76, Tr 2000, matrix 320270, Ti 130), T2-weighted (Tr 5000, Te 90, flip angle 150, matrix 512432), and T2 fluid attenuated inversion recovery (FLAIR) (Tr 9000, Te 93, flip angle 150, matrix 320*270, Ti 150). Each sequence had a slice thickness of 3 mm (Figure 4). The MRI revealed mild generalized cerebral atrophy and prominent cerebrospinal fluid (CSF) subdural spaces in the anterior temporal region, basifrontal area along the olfactory lobes, and left cerebellar hemisphere, along with atrophy of the olfactory bulb. Prominent Virchow-Robin (VR) spaces were observed in bilateral deep white matter, brainstem, and deep nuclei. Imaging also revealed craniosynostosis characterized by dolichocephaly, increased anteroposterior diameter, and reduced transverse diameter of the skull. These imaging features were consistent with mucopolysaccharidosis type VII (Sly syndrome).

## Discussion

Mucopolysaccharide disorders constitute a spectrum of rare, genetically inherited autosomal recessive diseases. These disorders typically manifest early in life with physical, skeletal, and developmental delays as well as neurological manifestations. The first neuroradiologic investigations in MPS were conducted using X-ray and computed tomography (CT). These revealed nonspecific imaging characteristics, notably subarachnoid and ventricular dilatation, and evidence of low-attenuation white matter regions [10].

Patients with MPS show a wide range of abnormalities on cranial and spinal magnetic resonance (MR) imaging. The most common brain abnormalities in almost all forms of the disease include alterations in white and gray matter, hydrocephalus, ventriculomegaly, cortical atrophy, and enlargement of the perivascular spaces (PVS). In contrast, spinal neuroimaging typically reveals canal stenosis, cord compression, and myelopathy [11]. An objective diagnostic method known as magnetic resonance spectroscopy (MR spectroscopy) measures the concentration of brain metabolites and provides information about potential relationships between these substances and underlying neuronal injury. Alterations in metabolites can be used to assess disease severity [11]. Using MR spectroscopy, Takahashi et al. [12] discovered a resonance greater than that of myo-inositol, linking this finding to in vitro glycosaminoglycans (GAGs) accumulation. Furthermore, they demonstrated an increased ratio of choline to creatine in white matter lesions, suggesting damage to the myelin sheath, increased glial proliferation, or increased cell membrane production.

Vedolin et al. investigated whether changes in MR spectroscopy could serve as a biomarker for cognitive decline in MPS patients. They discovered a larger myo-inositol/creatine ratio in cognitively impaired patients. They theorized that this MR spectroscopic observation was caused by GAG accumulation, increased astrocyte cell volume, or altered glial metabolism [13].

This article highlights a clinically and metabolically proven case of isolated mucopolysaccharidosis VII, also known as Sly syndrome, confirmed by increased levels of mucopolysaccharides, specifically dermatan sulfate, heparan sulfate, and chondroitin sulfate, in urine analysis. Consistent with existing literature, this case exhibits classical features such as dysostosis complex, including vertebral anomalies characterized by abnormal bullet-shaped cervical vertebrae, mild brain atrophy, and prominent VR spaces. However, the case also presented a range of previously undocumented neurological findings, including hypoplasia of the olfactory bulb, hypertelorism, a short mandibular body with abnormal angulation and hypoplasia of mandibular condyles, craniosynostosis with abnormal craniofacial bone shapes, and thickening of the frontal and occipital bones with external protuberance, expanding the spectrum of dysostosis multiplex.

Additionally, this case demonstrated spinal canal compromise at the vertebral junction, resulting in mild spinal cord compression due to abnormal vertebrae. Medical imaging played a crucial role in supporting the diagnosis of Sly syndrome and evaluating complications such as hydrocephalus, spinal cord compression, and brain atrophy.

## Conclusions

Although Sly syndrome (mucopolysaccharidosis VII) is a recognized but rare condition, its features can significantly overlap with mucopolysaccharidosis I (Hurler syndrome). This case report underscores the importance of recognizing both typical and atypical neurological features in Sly syndrome, which may initially present with nonspecific findings. Neuroimaging findings play a crucial role in facilitating early and accurate identification and diagnosis of Sly syndrome, including its complications. Early detection of this rare disorder can significantly improve the quality of life for affected patients.
